# Supplementary material for: Optical and electrical probing of plasmonic metal-molecule interactions
Source: Sci Adv. 2025 Dec 12;11(50):eaea0585. doi: 10.1126/sciadv.aea0585 (PMC12700213; doi:10.1126/sciadv.aea0585)
Supplement: Supplementary file 1 — Description of the theoretical model Tables S1 to S4 Figs. S1 to S9 Computational Details References [file sciadv.aea0585_sm.pdf]

Science Advances
AAAS

Supplementary Materials for

Optical and electrical probing of plasmonic metal-molecule interactions

Andrei Stefancu *et al.*

Corresponding author: Andrei Stefancu, andrei.stefancu@lmu.de; Emiliano Cortes, emiliano.cortes@lmu.de; Rupert Oulton, r.oulton@imperial.ac.uk; Wenxuan Tang, wenxuan.tang21@imperial.ac.uk

Sci. Adv. **11**, eaea0585 (2025)
DOI: 10.1126/sciadv.aea0585

This PDF file includes:

Description of the theoretical model
Tables S1 to S4
Figs. S1 to S9
Computational Details
References

Description of the theoretical model

The theoretical model of CID introduced by Persson considers the adsorbate damping of parallel, $\gamma_{\parallel}$, and perpendicular, $\gamma_{\perp}$ electric fields.

The parallel component, $\gamma_{\parallel}$, is characterized by an adsorbate-dependent electron scattering cross-section which, in the limit of plasmon frequency, $\hbar\omega_{SPP} \rightarrow 0$ yields the electron scattering cross-section for DC conditions (i.e., adsorbate-induced DC surface resistivity change). The adsorbate-dependent diffuse scattering cross-section can be computed as: (*32,34*)

$$\sigma_{diff}(\omega) = \sigma_0 J(\omega) \qquad \text{(S1)}$$

$$\sigma_0 = \frac{64\omega_F Q}{3\pi n v_F} \qquad \text{(S2)}$$

Where ω_F is the Fermi frequency, n is the density of metal electrons, v_F the Fermi velocity and Q is a number depending on the symmetry of the molecular orbital (0.33 for p orbitals and 0.2 for s orbitals).

At the plasmon resonance frequency, for CID, the $J(\omega)$ integral is given by:

$$J(\omega_{SPR}) = \frac{\pi}{4\hbar^2\omega_F}\frac{1}{\omega_{SPR}}\int_{E_F-\hbar\omega_{SPR}}^{E_F} dE[E\Gamma\rho(E+\hbar\omega_{SPR}) + (E+\hbar\omega_{SPR})\Gamma\rho(E)] \qquad \text{(S3)}$$

While for DC excitation (*i.e.*, $\omega = 0$) this becomes:

$$J(0) = \frac{\pi}{2}\Gamma\rho(E_F) \qquad \text{(S4)}$$

From this, we can calculate the change in the plasmon decay due to the damping of the parallel surface electric fields as $\Delta\gamma_{\parallel}[\text{Å } eV]$ and the DC scattering cross-section, Σ_{DC}:

$$\Delta\gamma_{\parallel} = {}^{3}/_{8R}\, v_F n_a \sigma_{diff}(\omega_{SPR}) \qquad \text{(S5)}$$

$$\Sigma_{DC} = \sigma_{diff}(0) \qquad \text{(S6)}$$

Likewise, the damping of normal surface electric fields, $\gamma_{\perp}$ due to adsorbates is:

$$\Delta\gamma_{\perp} = \frac{16\pi}{1+2\varepsilon_0} n_a \omega_{SPR} Im\alpha_{\perp}(\omega_{SPR}) \qquad \text{(S6)}$$

Where:

$$Im\alpha_{\perp}(\omega_{SPR}) = 2\pi(ed)^2 \int_{E_F-\hbar\omega_{SPR}}^{E_F} dE\ \rho(E)\rho(E+\hbar\omega_{SPR}) \qquad \text{(S7)}$$

Where d is the distance between the metal and the image plane of the molecular orbital.

The total CID rate can be written as:

$$\gamma_{CID} = \gamma_{\parallel} + \gamma_{\perp} \qquad \text{(S8)}$$

The total CID rate was calculated with a Python script adapted from ref. (*64,65*)

Table S1. Parameters used for calculating the CID and SR.

Parameter	Description	Value
n	Carrier concentration of core metal	0.059 $Å^{-3}$
E_F	Fermi energy of core metal	5.53 eV
Γ	Linewidth of adsorbate induced resonance or virtual state	1 eV
ε_0	Bulk dielectric constant of the adsorbate	2 (*66*)
Q	A number that mainly depends on the symmetry of the resonance state.	0.33 (for BPT) and 0.2 (for all other adsorbates)
d	Distance between dynamic image plane and center of mass of orbital	0.9 Å (*67*)
v_F	Fermi velocity of core metal	1.4×10^{-16} Å/s

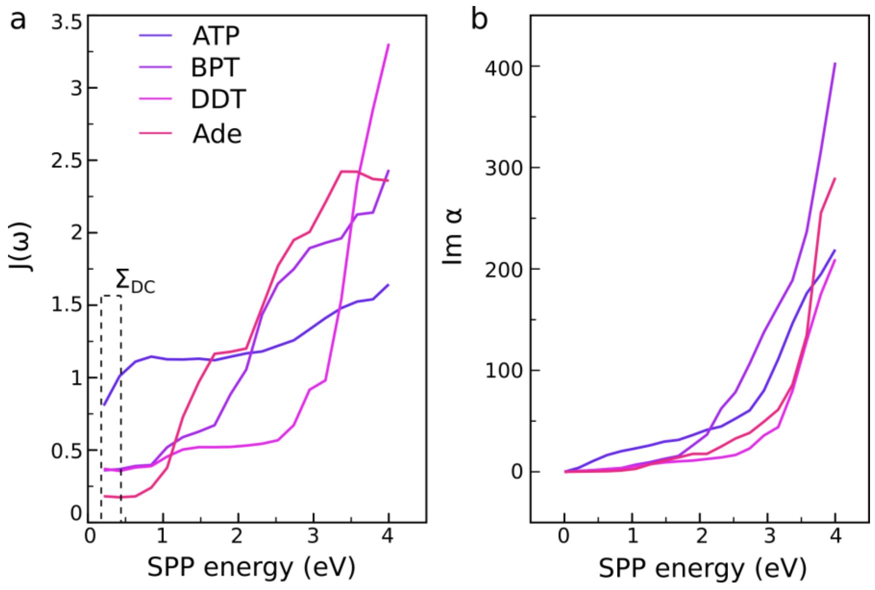

Figure S1. Calculated wavelength-dependent Chemical Interface Damping. The plasmon energy dependent a) $J(\omega)$ and b) $Im\ \alpha$ integrals for the four adsorbates. Note that, even if the integrals in the case of Adenine are large, the CID rate depends on the adsorbate concentration, n_a, which is smallest for Adenine compared to the other adsorbed molecules tested here.

Supplementary DC Surface Resistivity data

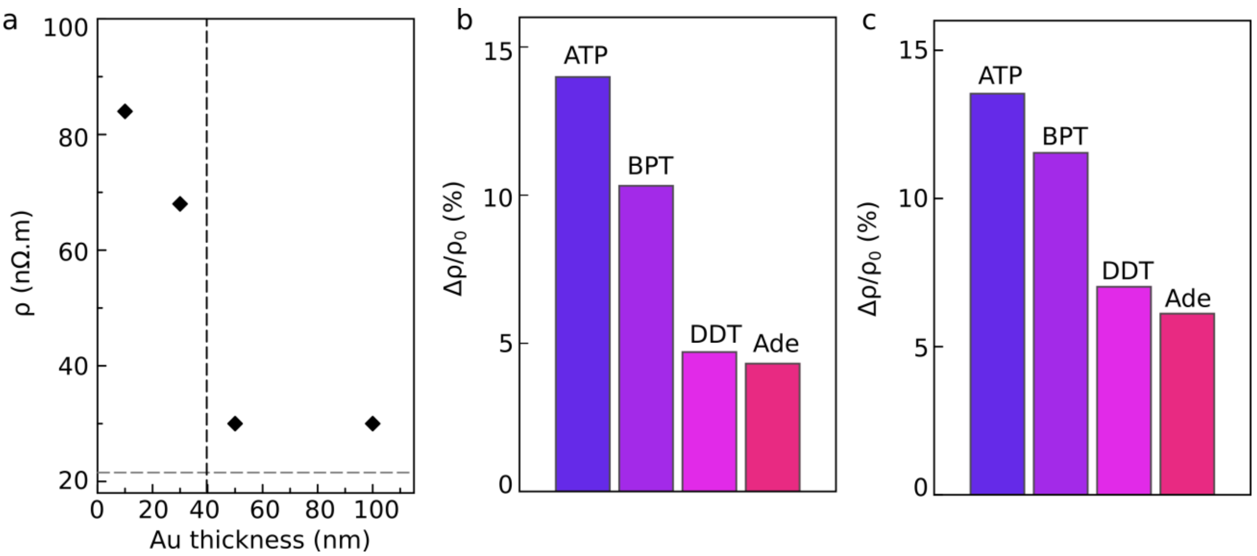

Figure S2. Adsorbate-induced change of surface resistivity. a) Resistivity of the blank Au films with different thickness. Relative change of resistivity of b) 30 and c) 15 nm thick Au films with the four different adsorbates upon overnight functionalization (1 mM concentration solutions).

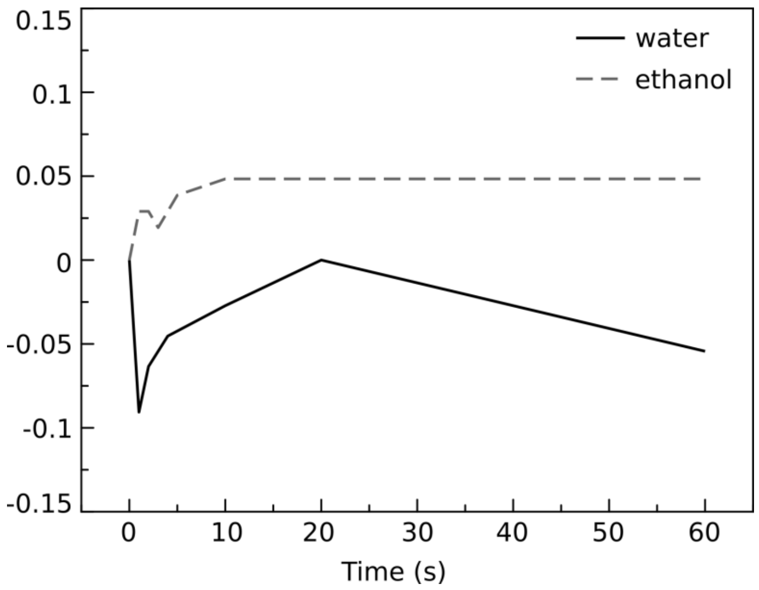

Figure S3. Solvent-induced change of surface resistivity. The relative change of Au film (30 nm) resistivity in time due to water and ethanol solvents only. Both solvents show a negligible change of resistivity. The slight decrease of resistivity for water is due to the solvation of physisorbed O atoms from the Au surface.

Supplementary CID data

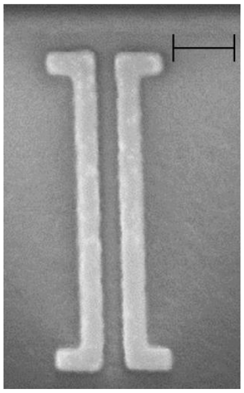

Figure S4. SEM image of the waveguide. The scale bar is 200 nm.

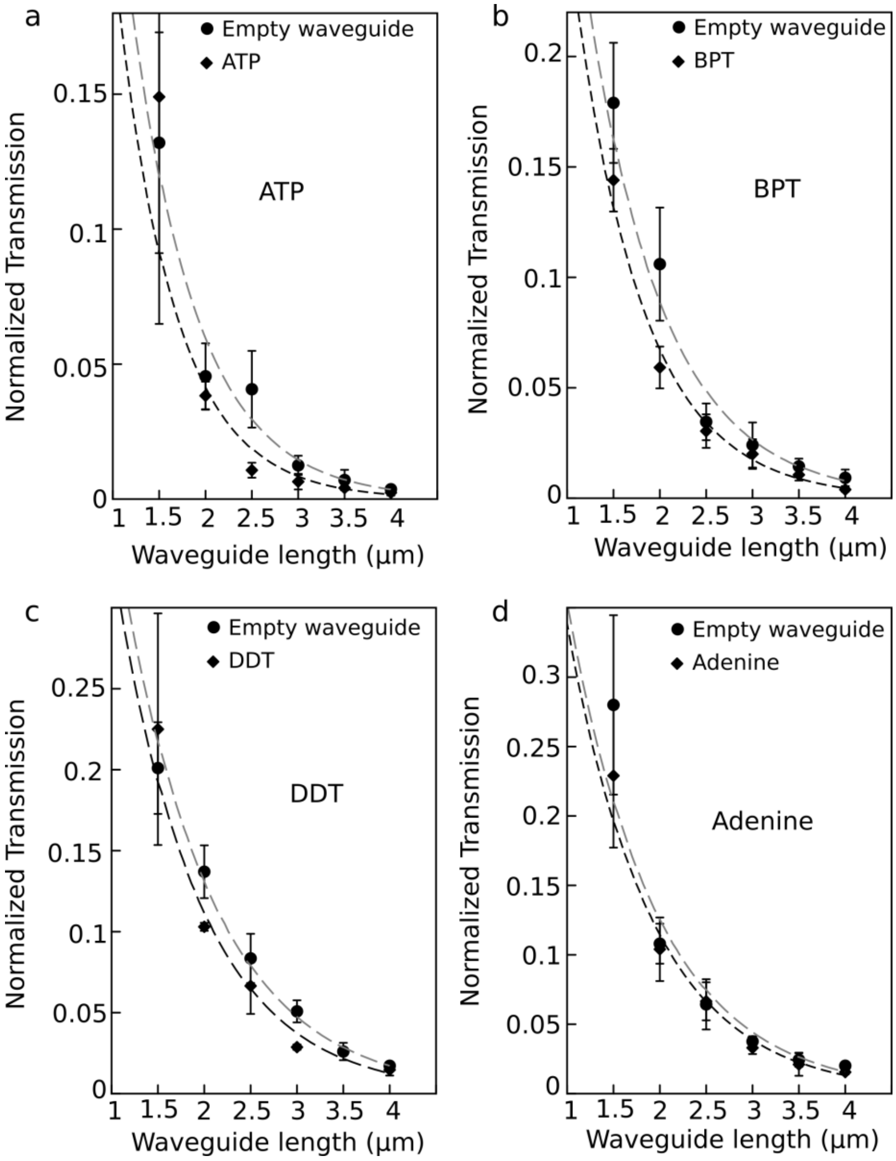

Figure S5. Plasmon propagation loss with different adsorbates. The total transmission of the waveguide versus the waveguide length with a) ATP, b) BPT, c) DDT and d) Ade molecular monolayers . The data is normalized based on the assumption that the antenna efficiency is unchanged after molecule deposition.

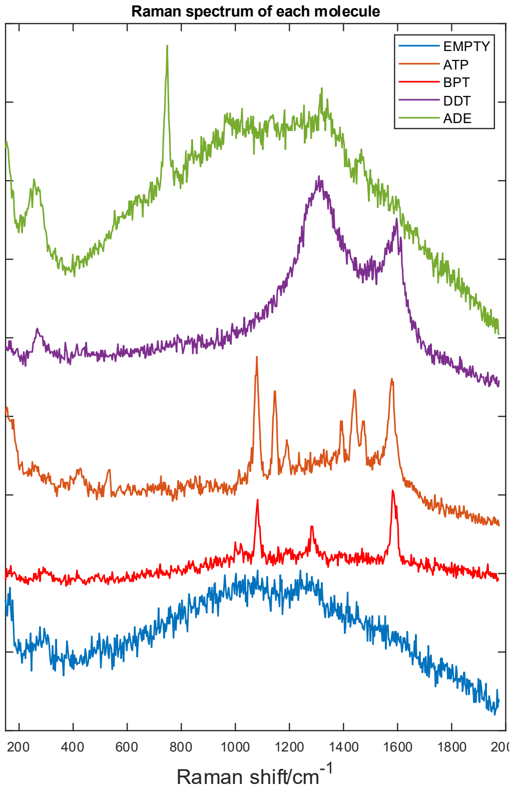

Figure S6. Raman verification of molecular functionalization. Raman spectrum of the empty plasmonic waveguide (blue spectrum) and the waveguide after molecular functionalization.

Table S2. Raw data for plasmon propagation loss on unfunctionalized waveguides and the waveguides with the four different adsorbates.

Molecules	L_{sp}[μm]	α [μm^{-1}]	η	$\alpha_{cid} = \Delta\alpha$ [μm^{-1}]	$\Delta\alpha/\alpha$	γ_{cid} [s^{-1}]
None	0.83 ± 0.19<br>0.71 ± 0.05<br>0.97 ± 0.03<br>0.96 ± 0.07*	1.20 ± 0.28<br>1.41 ± 0.10<br>1.03 ± 0.03<br>1.04 ± 0.08*	0.20 ± 0.01<br>0.16 ± 0.03<br>0.31 ± 0.03<br>0.14 ± 0.04	–	–	–
BPT	0.70 ± 0.09	1.43 ± 0.04	0.20 ± 0.01	0.23 ± 0.33	0.19 ± 0.28	2.6×10^{13}
ATP	0.63 ± 0.02	1.59 ± 0.05	0.16 ± 0.06	0.18 ± 0.11	0.12 ± 0.08	2.1×10^{13}
DDT	0.91 ± 0.02	1.10 ± 0.02	0.18 ± 0.04	0.07 ± 0.05	0.062 ± 0.003	8.1×10^{12}
ADE	0.91 ± 0.06	1.10 ± 0.07	0.20 ± 0.03	0.06 ± 0.10	0.05 ± 0.01	7.0×10^{12}

Dipole moment of adsorbed molecules

Table S3. Calculated dipole moment of the four molecules in gas phase.

**Gas Phase**	**μ_x (D)**	**μ_y (D)**	**μ_z (D)**	**μ_{Total} (D)**
ATP	-6.49894	0.96882	-1.52291	6.74493
BPT	-0.29990	0.25831	-4.61378	4.63073
ADE	-0.12221	2.35480	0.33608	2.38180
DDT	1.33521	-0.53598	-1.41358	2.01699

Table S4. Calculated dipole moment of the four molecules adsorbed on Au.

**Adsorbed**	**μ_x (D)**	**μ_y (D)**	**μ_z (D)**	**μ_{Total} (D)**
ATP	-1.97026	0.29265	-3.15089	3.72769
BPT	0.74087	0.17044	-5.68109	5.73173
ADE	0.25489	0.42529	-1.20447	1.30253
DDT	0.16619	-0.11374	-2.85415	2.86125

Computational Details

The four adsorbates we study are adenine (ADE), 4-aminothiophenol (ATP), biphenylthiol (BPT) and 1-dodecanethiol (DDT). We use a slab approach to model an Au (111) surface. The slab has 5 layers, a vacuum size of 10 *Å* and we construct a supercell of the slab.

For ADE and ATP we use a 3x3 supercell, and for BPT and DDT we use a $2\sqrt{3}\text{x}\sqrt{3}$ supercell, corresponding to different adsorbate densities. The geometry optimization was performed using the Vienna Ab Initio Simulation Package (VASP) with projector augmented waves (PAW) pseudopotentials and the PBE functional. (*68-72*) The plane wave cutoffs were chosen to be 420 *eV* for each structure. A Gaussian smearing scheme was used with a Gaussian broadening of 0.1 *eV*. Van der Waals corrections and dipole corrections were included. The Van der Waals correction was applied via the Grimme D3 dispersion correction. (*73-74*) All geometry optimizations were carried out at the Γ point, and the structures were relaxed to a force tolerance of 1 *eV/Å* per atom. The relaxed structures are shown in Fig. S7.

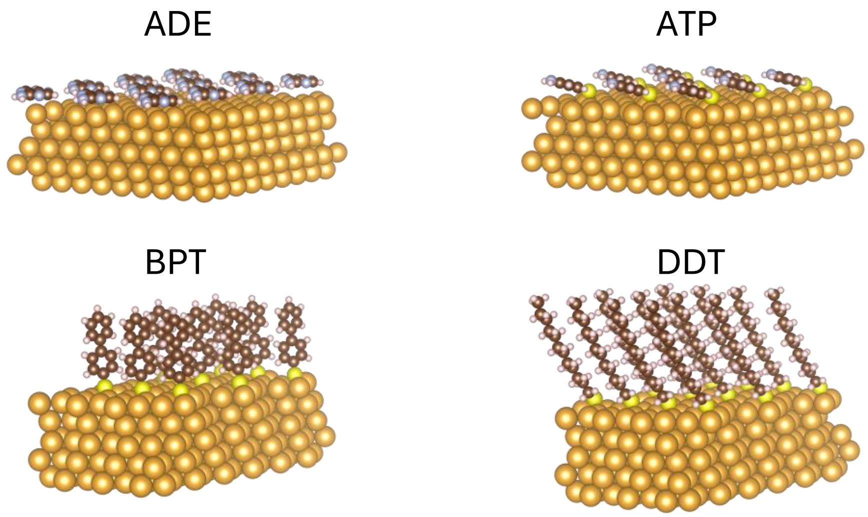

Figure S7. Molecular adsorption geometries. Relaxed structures of ADE, ATP, BPT and DDT adsorbed onto an Au (111) surface.

To evaluate the integrals from Persson's model, a density of states (DOS) must be obtained and projected onto the adsorbate.

Quantum Espresso was used for the density of states calculations. (*75-77*) GBRV ultrasoft pseudopotentials were used with a wavefunction cutoff of 40 *Ry* with a PBE functional. (*78*) A Gaussian smearing scheme was used with a broadening of 0.004 *Ry*. Both Grimme D3 Van der Waals dispersion correction and a dipole correction were applied. For the self-consistent field (SCF) cycles, a k-grid of 4x4x1 was used for each structure, and for the non self-consistent field cycles, a k-grid of 14x14x1 was used.

Persson's model uses a free electron gas model for Au. Therefore, a consistent choice for the Fermi energy that appears in Persson's integrals must be chosen. We choose to shift the DFT energies such that the Fermi energy for each structure agrees with the result from the free electron gas, i.e. $\varepsilon_F = \hbar^2(3\pi n)^{\frac{2}{3}}/m_e$,

where ε_F is the Fermi energy of the free electron gas, n is the number density of electrons and m_e is the mass of the electron.

To calculate the induced dipole moment in the metal due to the presence of the adsorbate, we use a cluster model of the Au (111) system.

To create the cluster, we start with the optimized periodic slab structures, taking one supercell and adding additional gold atoms around it. A cutoff radius is then chosen from a point centered on a gold atom directly below the adsorbate. All atoms that fall within this cutoff radius are included in the cluster, and all others are discarded. The remaining cluster is then centered in a 40x40x40 A° box. This procedure is shown diagrammatically in Fig. S8. The dipole moment is long ranged and so to capture the effects of the induced dipole moment a large surface area is needed. Thus, to keep computational costs down, only 3 layers of Au atoms are used in the cluster calculations.

To model the induced dipole moment due to the adsorbate, we take away the dipole moment of a pristine Au (111) cluster and the dipole moment of an isolated adsorbate away from the dipole moment of the Au (111) cluster with the adsorbate.

DFT calculations were performed on the clusters from Fig. S7 using the Vienna Ab Initio Simulation Package (VASP) with projector augmented waves (PAW) pseudopotentials and the PBE functional. The wavefunction cutoff was chosen to be 420 *eV* for each calculation. For calculations containing Au, a Gaussian smearing scheme was used with a Gaussian broadening of 0.1 *eV*. Both Grimme D3 Van der Waals dispersion correction and a dipole correction were applied to all calculations. All calculations were performed at the Γ point. The dipole moment was then obtained from the charge density.

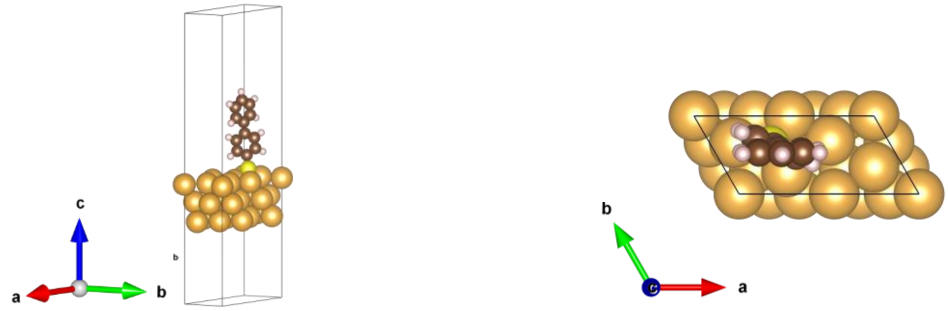

(a) Top view of optimized BPT periodic slab (b) Side view of optimized BPT periodic slab

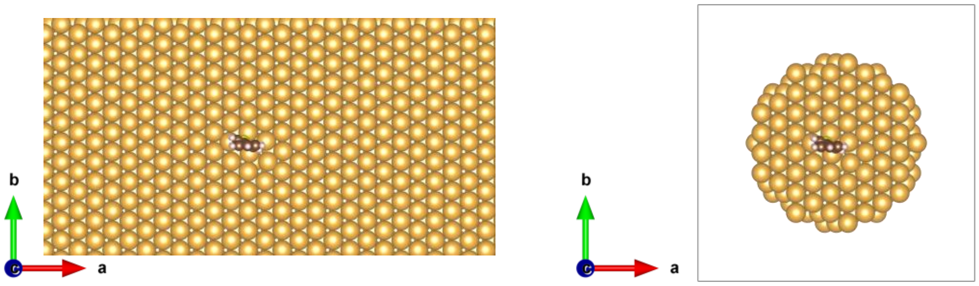

(c) Top view of optimized BPT periodic slab (d) Top view of cluster with extra Au atoms

Figure S8. **Calculation of the induced dipole moment of each molecule.** A relaxed periodic unit cell with one adsorbate (a, b) has Au atoms added to the boarders of the unit cell to produce (c). A defined cutoff radius is then specified to carve a cluster out of the system, e.g. a 12 *Å* radius was chosen to make (d).

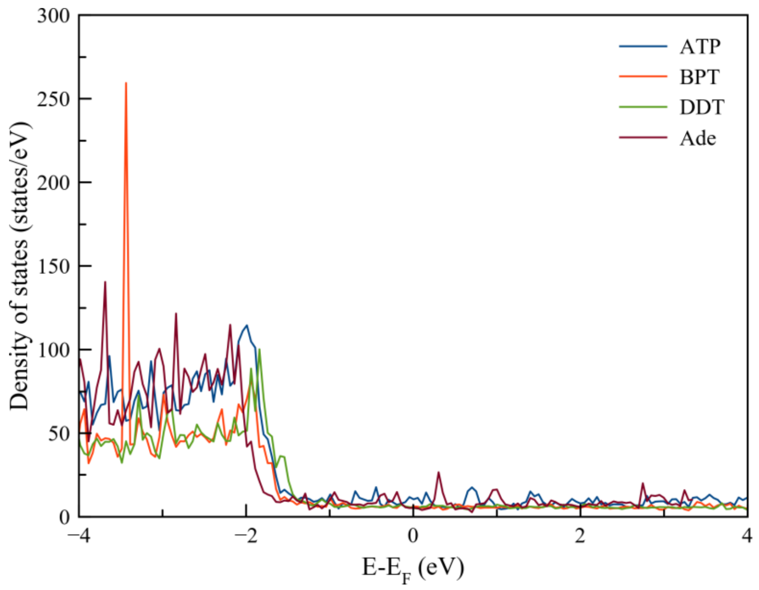

Figure S9. The metal density of states with the four different adsorbed molecules.

REFERENCES AND NOTES

1. Y. Dong, C. Hu, H. Xiong, R. Long, Y. Xiong, Plasmonic catalysis: New opportunity for selective chemical bond evolution. *ACS Catal.* **13**, 6730–6743 (2023).

2. M. L. Brongersma, N. J. Halas, P. Nordlander, Plasmon-induced hot carrier science and technology. *Nat. Nanotechnol.* **10**, 25–34 (2015).

3. A. Stefancu, N. J. Halas, P. Nordlander, E. Cortes, Electronic excitations at the plasmon–Molecule interface. *Nat. Phys.* **20**, 1065–1077 (2024).

4. L. Zhou, D. F. Swearer, C. Zhang, H. Robatjazi, H. Zhao, L. Henderson, L. Dong, P. Christopher, E. A. Carter, P. Nordlander, N. J. Halas, Quantifying hot carrier and thermal contributions in plasmonic photocatalysis. *Science* **362**, 69–72 (2018).

5. A. Stefancu, J. Gargiulo, G. Laufersky, B. Auguié, V. Chiş, E. C. le Ru, M. Liu, N. Leopold, E. Cortés, Interface-dependent selectivity in plasmon-driven chemical reactions. *ACS Nano* **17**, 3119–3127 (2023).

6. A. Stefancu, L. Nan, L. Zhu, V. Chiș, I. Bald, M. Liu, N. Leopold, S. A. Maier, E. Cortes, Controlling plasmonic chemistry pathways through specific ion effects. *Adv Opt Mater* **10**, 2200397 (2022).

7. A. Stefancu, S. Lee, L. Zhu, M. Liu, R. C. Lucacel, E. Cortés, N. Leopold, Fermi level equilibration at the metal–molecule interface in plasmonic systems. *Nano Lett.* **21**, 6592–6599 (2021).

8. C. F. Bohren, How can a particle absorb more than the light incident on it? *Am. J. Phys.* **51**, 323–327 (1983).

9. J. Jeong, H. H. Shin, Z. H. Kim, Unveiling the mechanism of plasmon photocatalysis via multiquantum vibrational excitation. *ACS Nano* **18**, 25290–25301 (2024).

10. G. N. Christenson, Z. Yu, R. R. Frontiera, Wavelength dependence of plasmon-induced vibrational energy transfer in fluorophore–plasmonic systems. *J. Phys. Chem. C* **128**, 10784–10789 (2024).

11. R. Sundararaman, P. Narang, A. S. Jermyn, W. A. Goddard III, H. A. Atwater, Theoretical predictions for hot-carrier generation from surface plasmon decay. *Nat. Commun.* **5**, 5788 (2014).

12. A. M. Brown, R. Sundararaman, P. Narang, W. A. Goddard III, H. A. Atwater, Nonradiative plasmon decay and hot carrier dynamics: Effects of phonons, surfaces, and geometry. *ACS Nano* **10**, 957–966 (2016).

13. J. Li, S. K. Cushing, F. Meng, T. R. Senty, A. D. Bristow, N. Wu, Plasmon-induced resonance energy transfer for solar energy conversion. *Nat. Photonics* **9**, 601–607 (2015).

14. X. You, S. Ramakrishna, T. Seideman, Unified theory of plasmon-induced resonance energy transfer and hot electron injection processes for enhanced photocurrent efficiency. *J. Chem. Phys.* **149**, 174304 (2018).

15. J. Khurgin, A. Y. Bykov, A. V. Zayats, Hot-electron dynamics in plasmonic nanostructures: Fundamentals, applications and overlooked aspects. *eLight* **4**, 15 (2024).

16. H. Petek, Photoexcitation of adsorbates on metal surfaces: One-step or three-step. *J. Chem. Phys.* **137**, 091704 (2012).

17. P. Avouris, R. E. Walkup, A. R. Rossi, H. C. Akpati, P. Nordlander, T. C. Shen, G. C. Abeln, J. W. Lyding, Breaking individual chemical bonds via STM-induced excitations. *Surf. Sci.* **363**, 368–377 (1996).

18. T. C. Shen, C. Wang, G. C. Abeln, J. R. Tucker, J. W. Lyding, P. Avouris, R. E. Walkup, Atomic-scale desorption through electronic and vibrational excitation mechanisms. *Science* **268**, 1590–1592 (1995).

19. A. Stefancu, O. M. Biro, O. Todor-Boer, I. Botiz, E. Cortés, N. Leopold, Halide–metal complexes at plasmonic interfaces create new decay pathways for plasmons and excited molecules. *ACS Photonics* **9**, 895–904 (2022).

20. B. Foerster, A. Joplin, K. Kaefer, S. Celiksoy, S. Link, C. Sönnichsen, Chemical interface damping depends on electrons reaching the surface. *ACS Nano* **11**, 2886–2893 (2017).

21. S. Tan, Y. Dai, S. Zhang, L. Liu, J. Zhao, H. Petek Coherent electron transfer at the Ag/graphite heterojunction interface. *Phys. Rev. Lett.* **120**, 126801 (2018).

22. B. Foerster, V. A. Spata, E. A. Carter, C. Sönnichsen, S. Link, Plasmon damping depends on the chemical nature of the nanoparticle interface. *Sci. Adv.* **5**, eaav0704 (2019).

23. Q. Zhang, W. Li, R. Zhao, P. Tang, J. Zhao, G. Wu, X. Chen, M. Hu, K. Yuan, J. Li, X. Yang, Real-time observation of two distinctive non-thermalized hot electron dynamics at MXene/molecule interfaces. *Nat. Commun.* **15**, 4406 (2024).

24. B. N. J. Persson, K. Zhao, Z. Zhang, Chemical contribution to surface-enhanced raman scattering. *Phys. Rev. Lett.* **96**, 207401 (2006).

25. T. Christensen, W. Yan, A. P. Jauho, M. Soljačić, N. A. Mortensen, Quantum corrections in nanoplasmonics: Shape, scale, and material. *Phys. Rev. Lett.* **118**, 157402 (2017).

26. L. Zurak, C. Wolff, J. Meier, R. Kullock, N. A. Mortensen, B. Hecht, T. Feichtner, Modulation of surface response in a single plasmonic nanoresonator. *Sci. Adv.* **10**, eadn5227 (2024).

27. R. G. Tobin, Mechanisms of adsorbate-induced surface resistivity—Experimental and theoretical developments. *Surf. Sci.* **502-503**, 374–387 (2002).

28. P. Shi, J. Zhang, H. Y. Lin, P. W. Bohn, Effect of molecular adsorption on the electrical conductance of single Au nanowires fabricated by electron-beam lithography and focused Ion beam etching. *Small* **6**, 2598–2603 (2010).

29. T. Kato, T. Tanaka, T. Yajima, K. Uchida, Temperature dependence of resistivity increases induced by thiols adsorption in gold nanosheets. *Jpn. J. Appl. Phys.* **60**, SBBH13 (2021).

30. A. Otto, P. Lilie, P. Dumas, C. Hirschmugl, M. Pilling, G. P. Williams, Anisotropic electric surface resistance of Cu(110). *New J. Phys.* **9**, 288 (2007).

31. B. N. J. Persson, A. I. Volokitin, Infrared reflection-absorption spectroscopy of dipole-forbidden adsorbate vibrations. *Surf. Sci.* **310**, 314–336 (1994).

32. B. N. J. Persson, Polarizability of small spherical metal particles: Influence of the matrix environment. *Surf. Sci.* **281**, 153–162 (1993).

33. E. Pensa, E. Cortés, G. Corthey, P. Carro, C. Vericat, M. H. Fonticelli, G. Benítez, A. A. Rubert, R. C. Salvarezza, The chemistry of the sulfur–gold interface: In search of a unified model. *Acc. Chem. Res.* **45**, 1183–1192 (2012).

34. B. N. J. Persson, Surface resistivity and vibrational damping in adsorbed layers. *Phys. Rev. B* **44**, 3277–3296 (1991).

35. H. Grabhorn, A. Otto, D. Schumacher, B. N. J. Persson, Variation of the DC-resistance of smooth and atomically rough silver films during exposure to C_2H_6 and C_2H_4. *Surf. Sci.* **264**, 327–340 (1992).

36. C. Vericat, M. E. Vela, G. Benitez, P. Carro, R. C. Salvarezza, Self-assembled monolayers of thiols and dithiols on gold: New challenges for a well-known system. *Chem. Soc. Rev.* **39**, 1805–1834 (2010).

37. B. I. Rosario-Castro, E. R. Fachini, J. Hernández, M. E. Pérez-Davis, C. R. Cabrera, Electrochemical and surface characterization of 4-aminothiophenol adsorption at polycrystalline platinum electrodes. *Langmuir* **22**, 6102–6108 (2006).

38. H. Hinterwirth, S. Kappel, T. Waitz, T. Prohaska, W. Lindner, M. Lämmerhofer, Quantifying thiol ligand density of self-assembled monolayers on gold nanoparticles by inductively coupled plasma–mass spectrometry. *ACS Nano* **7**, 1129–1136 (2013).

39. R. G. Acres, X. Cheng, K. Beranová, S. Bercha, T. Skála, V. Matolín, Y. Xu, K. C. Prince, N. Tsud, An experimental and theoretical study of adenine adsorption on Au(111). *Phys. Chem. Chem. Phys.* **20**, 4688–4698 (2018).

40. B. N. J. Persson, Applications of surface resistivity to atomic scale friction, to the migration of "hot" adatoms, and to electrochemistry. *J. Chem. Phys.* **98**, 1659–1672 (1993).

41. I. M. Ikram, M. K. Rabinal, Short chain molecular junctions: Charge transport versus dipole moment. *Appl. Surf. Sci.* **332**, 181–185 (2015).

42. D. A. Egger, E. Zojer, Anticorrelation between the evolution of molecular dipole moments and induced work function modifications. *J. Phys. Chem. Lett.* **4**, 3521–3526 (2013).

43. T. C. Leung, C. L. Kao, W. S. Su, Y. J. Feng, C. T. Chan, Relationship between surface dipole, work function and charge transfer: Some exceptions to an established rule. *Phys. Rev. B* **68**, 195408 (2003).

44. J. Chen, S. Gathiaka, Z. Wang, M. Thuo, Role of molecular dipoles in charge transport across large area molecular junctions delineated using isomorphic self-assembled monolayers. *J. Phys. Chem. C* **121**, 23931–23938 (2017).

45. M. Fu, M. P. d. P. Mota, X. Xiao, A. Jacassi, N. A. Güsken, Y. Chen, H. Xiao, Y. Li, A. Riaz, S. A. Maier, R. F. Oulton, Near-unity Raman β-factor of surface-enhanced Raman scattering in a waveguide. *Nat. Nanotechnol.* **17**, 1251–1257 (2022).

46. J. Bhandari, G. V. Hartland, Energy transfer for leaky surface plasmon polaritons in gold nanostripes. *J. Phys. Chem. C* **129**, 535–541 (2025).

47. B. S. Brown, G. V. Hartland, Chemical interface damping for propagating surface plasmon polaritons in gold nanostripes. *J. Chem. Phys.* **152**, 024707 (2020).

48. Y. Okamura, S. Yoshinaka, S. Yamamoto, Measuring mode propagation losses of integrated optical waveguides: A simple method. *Appl. Optics* **22**, 3892–3894 (1983).

49. S. Tan, A. Argondizzo, J. Ren, L. Liu, J. Zhao, H. Petek, Plasmonic coupling at a metal/semiconductor interface. *Nat. Photonics* **11**, 806–812 (2017).

50. S. Tan, L. Liu, Y. Dai, J. Ren, J. Zhao, H. Petek, Ultrafast plasmon-enhanced hot electron generation at Ag nanocluster/graphite heterojunctions. *J. Am. Chem. Soc.* **139**, 6160–6168 (2017).

51. S. P. Rittmeyer, J. Meyer, K. Reuter, Nonadiabatic vibrational damping of molecular adsorbates: Insights into electronic friction and the role of electronic coherence. *Phys. Rev. Lett.* **119**, 176808 (2017).

52. S. Kumar, H. Jiang, M. Schwarzer, A. Kandratsenka, D. Schwarzer, A. M. Wodtke, Vibrational relaxation lifetime of a physisorbed molecule at a metal surface. *Phys. Rev. Lett.* **123**, 156101 (2019).

53. J. Li, H. Qian, H. Chen, Z. Zhao, K. Yuan, G. Chen, A. Miranda, X. Guo, Y. Chen, N. Zheng, M. S. Wong, J. Zheng, Two distinctive energy migration pathways of monolayer molecules on metal nanoparticle surfaces. *Nat. Commun.* **7**, 10749 (2016).

54. B. N. J. Persson, A. I. Volokitin, Electronic friction of physisorbed molecules. *J. Chem. Phys.* **103**, 8679–8683 (1995).

55. W. Dou, J. E. Subotnik, Perspective: How to understand electronic friction. *J. Chem. Phys.* **148**, 230901 (2018).

56. M. Head-Gordon, J. C. Tully, Molecular dynamics with electronic frictions. *J. Chem. Phys.* **103**, 10137–10145 (1995).

57. A. I. Volokitin, B. N. J. Persson, Quantum theory of infrared-reflection spectroscopy from adsorbate-covered metal surfaces in the anomalous-skin-effect frequency region. *Phys. Rev. B* **52**, 2899–2906 (1995).

58. M. V.- F. Pfeiffer, G. B. Vonbun-Feldbauer, J. B. Khurgin, O. Matts, A. Shqer, N. Mameka, M. Eich, A. Petrov, Energy dependent chemical interface damping induced by 1-decanethiol self-assembled monolayer on Au(111). arXiv:2509.05025 [cond-mat.mtrl-sci] (2025).

59. F. Fassioli, R. Dinshaw, P. C. Arpin, G. D. Scholes, Photosynthetic light harvesting: Excitons and coherence. *J. R. Soc. Interface* **11**, 20130901 (2014).

60. S. Wang, G. D. Scholes, L. Y. Hsu, Coherent-to-incoherent transition of molecular fluorescence controlled by surface plasmon polaritons. *J. Phys. Chem. Lett.* **11**, 5948–5955 (2020).

61. S. R. Rather, G. D. Scholes, From fundamental theories to quantum coherences in electron transfer. *J. Am. Chem. Soc.* **141**, 708–722 (2019).

62. E. N. Zimanyi, R. J. Silbey, Unified treatment of coherent and incoherent electronic energy transfer dynamics using classical electrodynamics. *J. Chem. Phys.* **133**, 144107 (2010).

63. M. Brandbyge, P. Hedegård, T. F. Heinz, J. A. Misewich, D. M. Newns, Electronically driven adsorbate excitation mechanism in femtosecond-pulse laser desorption. *Phys. Rev. B* **52**, 6042–6056 (1995).

64. T. Yuan, X. Guo, S. A. Lee, S. Brasel, A. Chakraborty, D. J. Masiello, S. Link, Chemical interface damping revealed by single-particle absorption spectroscopy. *ACS Nano* **19**, 10277–10288 (2025).

65. B. Ostovar, S. A. Lee, A. Mehmood, K. Farrell, E. K. Searles, B. Bourgeois, W.-Y. Chiang, A. Misiura, N. Gross, A. Al-Zubeidi, J. A. Dionne, C. F. Landes, M. Zanni, B. G. Levine, S. Link, The role of the plasmon in interfacial charge transfer. *Sci. Adv.* **10**, eadp3353 (2024).

66. H. B. Akkerman, R. C. G. Naber, B. Jongbloed, P. A. van Hal, P. W. M. Blom, D. M. de Leeuw, B. de Boer, Electron tunneling through alkanedithiol self-assembled monolayers in large-area molecular junctions. *Proc. Natl. Acad. Sci. U.S.A.* **104**, 11161–11166 (2007).

67. Z.-F. Liu, D. A. Egger, S. Refaely-Abramson, L. Kronik, J. B. Neaton, Energy level alignment at molecule-metal interfaces from an optimally tuned range-separated hybrid functional. *J. Chem. Phys.* **146**, 092326 (2017).

68. G. Kresse, J. Furthmüller, Efficiency of ab-initio total energy calculations for metals and semiconductors using a plane-wave basis set. *Comput. Mater. Sci.* **6**, 15–50 (1996).

69. G. Kresse, J. Furthmüller, Efficient iterative schemes forab initiototal-energy calculations using a plane-wave basis set. *Phys. Rev. B* **54**, 11169–11186 (1996).

70. G. Kresse, D. Joubert, From ultrasoft pseudopotentials to the projector augmented-wave method. *Phys. Rev. B* **59**, 1758–1775 (1999).

71. G. Kresse, J. Hafner, Ab initio molecular dynamics for liquid metals. *Phys. Rev. B* **47**, 558–561 (1993).

72. J. P. Perdew, K. Burke, M. Ernzerhof, Generalized gradient approximation made simple. *Phys. Rev. Lett.* **77**, 3865–3868 (1996).

73. L. Bengtsson, Dipole correction for surface supercell calculations. *Phys. Rev. B* **59**, 12301–12304 (1999).

74. S. Grimme, J. Antony, S. Ehrlich, H. Krieg, A consistent and accurate ab initio parametrization of density functional dispersion correction (DFT-D) for the 94 elements H-Pu. *J. Chem. Phys.* **132**, 154104 (2010).

75. P. Giannozzi, O. Baseggio, P. Bonfà, D. Brunato, R. Car, I. Carnimeo, C. Cavazzoni, S. de Gironcoli, P. Delugas, F. Ferrari Ruffino, A. Ferretti, N. Marzari, I. Timrov, A. Urru, S. Baroni, Quantum ESPRESSO toward the exascale. *J. Chem. Phys.* **152**, 154105 (2020).

76. P. Giannozzi, S. Baroni, N. Bonini, M. Calandra, R. Car, C. Cavazzoni, D. Ceresoli, G. L. Chiarotti, M. Cococcioni, I. Dabo, A. Dal Corso, S. de Gironcoli, S. Fabris, G. Fratesi, R. Gebauer, U. Gerstmann, C. Gougoussis, A. Kokalj, M. Lazzeri, L. Martin-Samos, N. Marzari, F. Mauri, R. Mazzarello, S. Paolini, A. Pasquarello, L. Paulatto, C. Sbraccia, S. Scandolo, G.

Sclauzero, A. P. Seitsonen, A. Smogunov, P. Umari, R. M. Wentzcovitch, QUANTUM ESPRESSO: A modular and open-source software project for quantum simulations of materials. *J. Phys. Condens. Matter* **21**, 395502 (2009).

77. P. Giannozzi, O. Andreussi, T. Brumme, O. Bunau, M. Buongiorno Nardelli, M. Calandra, R. Car, C. Cavazzoni, D. Ceresoli, M. Cococcioni, N. Colonna, I. Carnimeo, A. Dal Corso, S. de Gironcoli, P. Delugas, R. A. DiStasio Jr., A. Ferretti, A. Floris, G. Fratesi, G. Fugallo, R. Gebauer, U. Gerstmann, F. Giustino, T. Gorni, J. Jia, M. Kawamura, H. Y. Ko, A. Kokalj, E. Küçükbenli, M. Lazzeri, M. Marsili, N. Marzari, F. Mauri, N. L. Nguyen, H. V. Nguyen, A. Otero-de-la-Roza, L. Paulatto, S. Poncé, D. Rocca, R. Sabatini, B. Santra, M. Schlipf, A. P. Seitsonen, A. Smogunov, I. Timrov, T. Thonhauser, P. Umari, N. Vast, X. Wu, S. Baroni, Advanced capabilities for materials modelling with Quantum ESPRESSO. *J. Phys. Condens. Matter* **29**, 465901 (2017).

78. K. F. Garrity, J. W. Bennett, K. M. Rabe, D. Vanderbilt, Pseudopotentials for high-throughput DFT calculations. *Comput. Mater. Sci.* **81**, 446–452 (2014).
